# Tailoring tobacco hairy root metabolism for the production of stilbenes

**DOI:** 10.1038/s41598-017-18330-w

**Published:** 2017-12-21

**Authors:** Diego Hidalgo, Milen Georgiev, Andrey Marchev, Roque Bru-Martínez, Rosa M. Cusido, Purificación Corchete, Javier Palazon

**Affiliations:** 1 Laboratori de Fisiologia Vegetal, Facultat de Farmacia, Universitat de Barcelona, 08028 Barcelona, Spain; 2 Group of Plant Cell Biotechnology and Metabolomics, The Stephan Angeloff Institute of Microbiology, Bulgarian Academy of Sciences, Plovdiv, 4000 Bulgaria; 3 Plant Proteomics and Functional Genomics Group, Department of Agrochemistry and Biochemistry, Faculty of Science, University of Alicante, Alicante, Spain; 4 Department of Plant Physiology, Campus Miguel de Unamuno, University of Salamanca, E-37007 Salamanca, Spain

## Abstract

Tobacco hairy root (HR) cultures, which have been widely used for the heterologous production of target compounds, have an innate capacity to bioconvert exogenous *t*-resveratrol (*t*-R) into *t-*piceatannol (*t*-Pn) and *t*-pterostilbene (*t*-Pt). We established genetically engineered HR carrying the gene encoding stilbene synthase (STS) from *Vitis vinifera* and/or the transcription factor (TF) AtMYB12 from *Arabidopsis thaliana*, in order to generate a holistic response in the phenylpropanoid pathway and coordinate the up-regulation of multiple metabolic steps. Additionally, an artificial microRNA for chalcone synthase (amiRNA CHS) was utilized to arrest the normal flux through the endogenous chalcone synthase (CHS) enzyme, which would otherwise compete for precursors with the STS enzyme imported for the flux deviation. The transgenic HR were able to biosynthesize the target stilbenes, achieving a production of 40 μg L^−1^ of *t*-R, which was partially metabolized into *t*-Pn and *t*-Pt (up to 2.2 μg L^−1^ and 86.4 μg L^−1^, respectively), as well as its glucoside piceid (up to 339.7 μg L^−1^). Major metabolic perturbations were caused by the TF AtMYB12, affecting both primary and secondary metabolism, which confirms the complexity of biotechnological systems based on seed plant *in vitro* cultures for the heterologous production of high-value molecules.

## Introduction

Due to its wide-ranging therapeutic potential, resveratrol (3,5,4′-trihydroxy-trans-stilbene) (*t*-R) is amongst the most studied stilbenes. Its antiviral, antioxidant, anti-inflammatory, and cardioprotective effects have been extensively reported, as well as platelet anti-aggregation and melanoma chemoprevention activities^1^. The hydroxylated *t*-R derivative *t*-piceatannol (*t*-Pn) has significant anti-cancer and cancer chemopreventive activity but with higher bioavailability than *t*-R^2,3^, while the methoxy derivative *t*-pterostilbene (*t*-Pt) has greater anti-proliferative effects against human colon cancer cells^4^. The scarce distribution of these derivatives in nature calls for the development of alternative sources for their sustainable supply. In this scenario, biotechnological factories based on plant cell cultures (the so-called “green cell factory” concept) constitute a promising new biosustainable system for plant secondary metabolite (PSM) production. Similarly, hairy root (HR) cultures hold great potential as systems for the bioconversion and bioproduction of PSM^5^, due to their genetic stability^6,7^ and facile scale up to bioreactor level^8^. Additionally, HR do not need exogenous hormones and allow the large-scale harvesting of secondary products^9^, in contrast with plant cell cultures, which are frequently unable to produce significant levels of target compounds^8^. The use of HR as biofactories has been reviewed by Chandra and Chandra^10^ and Rischer *et al*.^11^.

The heterologous biosynthesis of *t*-R has been reported in microorganisms and whole plants^12,13^. Recently, this strategy has been successfully used in metabolically engineered grapevine cell cultures to obtain the highly bioactive stilbenes, *t*-Pn and *t*-Pt^14^. The capacity of engineered tobacco HR cultures to bioconvert *t*-R into the target derivatives has also been demonstrated^15^. In addition, biotechnological studies have shown that *Vitis vinifera* cell cultures need elicitation treatments for an efficient *t*-R production, the best results being achieved by combining methyl jasmonate with the expensive cyclodextrins^16^. *Silybum marianum* transgenic cell suspensions carrying the *V.vinifera* stilbene synthase3 (*Vv*STS) gene treated only with cyclodextrins have also yielded *t*-R^17^, as have HR of peanut^18,19^ and *V. rotundifolia* ^20^.

Metabolic engineering has been used to enhance biosynthetic pathways in plants^21,22^, extending existing routes or introducing new ones to obtain novel compounds^10,23,24^. Approaches based on the modification of single steps have certain drawbacks, such as the inability to address multiple bottlenecks or simultaneously target multiple metabolites. Consequently, more effective approaches have been developed involving the overexpression of transcription factors (TF) that can simultaneously regulate multiple genes in a metabolic pathway^25^. On the other hand, the use of omics technologies has led to significant advances in the elucidation of secondary metabolism and has shed light on the competition between primary and secondary pathways^26,27^. In most cases TF are present as gene families and can directly activate all or several of the enzymes involved in metabolite formation in response to biotic and abiotic stress^28,29^. Moreover, TF allow gaps or undefined stages in metabolic routes to be reduced or eliminated upon transfer to heterologous systems^30^. The MYB TF family is the most useful in enhancing flavonoid biosynthesis^31,32^, when expressed in tobacco and tomato. Notably, AtMYB12 TF increased the levels of phenolic compounds and insect resistance^33,34^. Recent studies have reported strong modulation in transcriptome behavior by MYB TF, which above all affects genes of the phenylpropanoid pathway^35^. In strategies aimed at increasing the production of a specific metabolite, the insertion of a single gene to complete the desired metabolic pathway can be more effective when accompanied by a TF-mediated holistic modification^34^.

The blocking of competitive pathways is another successfully used approach in metabolic engineering to improve PSM production. In artificial micro RNA (amiRNA) technology, which involves endogenous primary-miRNAs (pri-miRNAs), the miRNA and miRNA* sequences are replaced with corresponding artificial-miRNA (amiRNA/amiRNA*) sequences for the specific target gene^36^. The application of amiRNA in plants has led to the elucidation of gene function in the phenylpropanoid pathway^34,35^, as well as the identification of genes involved in plant growth and development^37–41^. Recently, Carbonell *et al*.^42^ developed a relatively straightforward protocol for the fast and effective construction of amiRNAs for plant species, which opens up new possibilities for research in plant biotechnology.

**Figure 1:**
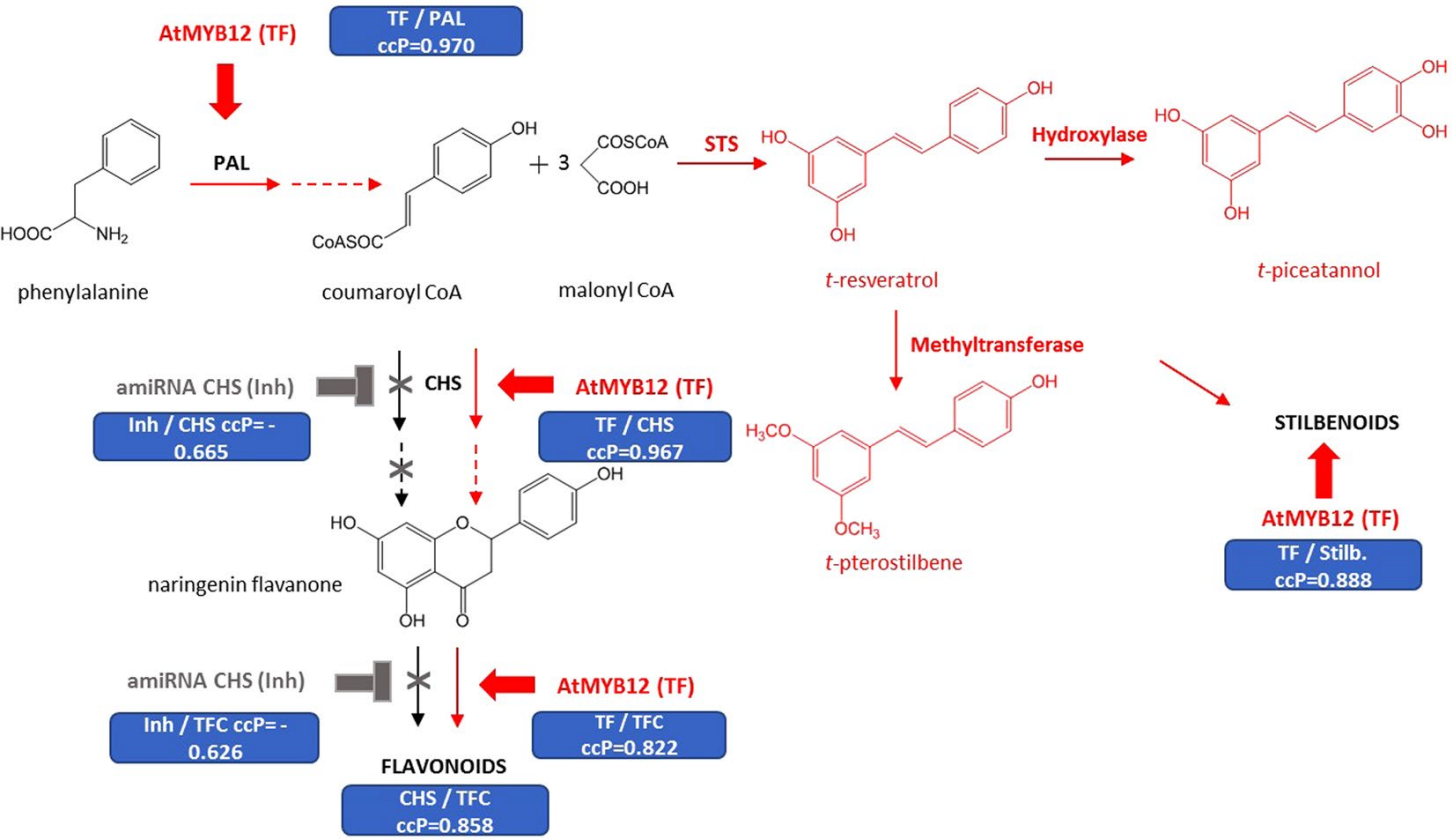
Metabolic perturbations of the phenolic biosynthetic pathway in the transgenic tobacco HR clones caused by heterologous gene expression. PAL, phenylalanine ammonia lyase; STS, stilbene synthase; CHS, chalcone synthase; TFC, total flavonoid content; Stilb, total stilbene contents; Inh, amiRNA-CHS expression; TF, AtMYB12 TF expression; In red, activated pathway; In grey, inhibited pathway; Blue box, value of the mathematical correlation between gene expression and/or plant secondary metabolite accumulation; ccP, Pearson correlation coefficient.

In the present work, we report the engineering of tobacco HR carrying the VvSTS gene to tailor the HR metabolism for the bioproduction of *t*-R and its derivatives *t*-Pn and *t*-Pt. Thus, the AtMYB12 TF was overexpressed to generate a holistic response in the phenylpropanoid pathway and coordinate the up-regulation of multiple steps. In addition, the use of artificial microRNA for chalcone synthase (amiRNA-CHS) disrupted the normal flux through the endogenous CHS enzyme, which competes for precursors with the imported stilbene synthase (STS) enzyme, thereby promoting the formation of *t*-R (Fig. 1).

## Results

### Establishment and genetic characterization of transgenic tobacco root cultures

**Figure 2:**
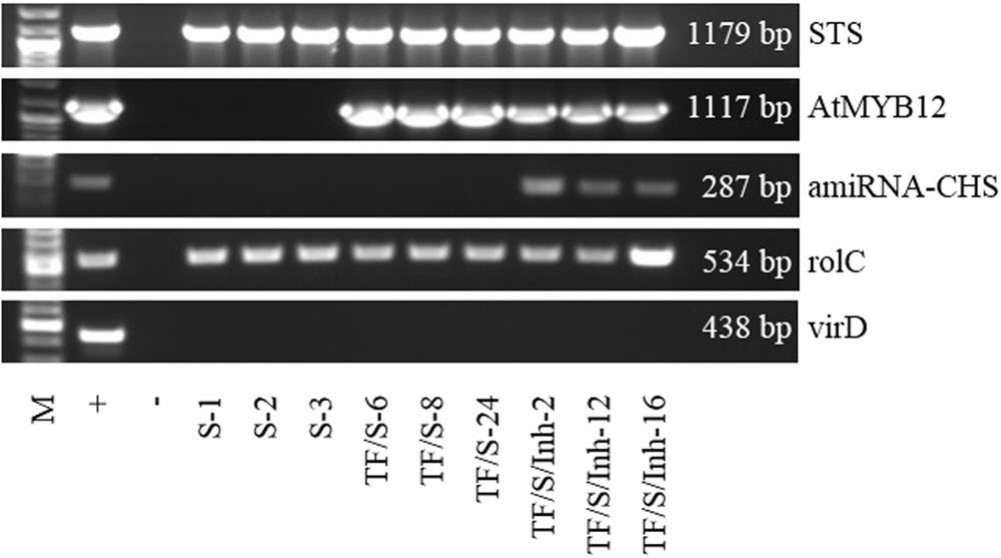
PCR analysis of the genomic DNA of HR lines. (+) positive control (corresponding *A. rhizogenes* used for the infections), (−) negative control (DNA of control HR or DNA of *Nicotiana tabacum* wild type plant for *rolC*). The positions S-1 to TF/S/Inh-16 represent the analyzed HR lines showing the presence of the corresponding transgenes. The full-length gels are included in a Supplementary material.

Transgenic tobacco HR cultures were obtained by co-culture of leaf segments from *in vitro* tobacco plantlets with a wild type *Agrobacterium rhizogenes* A4 (pRiA4) strain (control line) or the engineered bacteria *A. rhizogenes* containing the plasmids [pRiA4 + pJCV52-STS3], [PRiA4 + pJCV52-AtMYB12] and [pRiA4 + pDMDC32B-AtMiR390a-B/c amiRNACHS]. The HR appeared at 2–4 weeks, after which they were cultured individually in MS solid medium, supplemented with cefotaxime (500 mg L^−1^) and kanamycin (100 mg L^−1^) and/or hygromycin (50 mg L^−1^), depending on the target gene. Most of the transgenic root lines isolated after the infection with *A. rhizogenes* A4-S or co-infection with A4-S and A4-TF grew well under kanamycin selection, whereas only 40% of the root lines that appeared after the co-infection with the Agrobacteria A4-S, A4-TF and A4-Inh survived under joint kanamycin and hygromycin selection. After successive 2-week subcultures, the antibiotic concentration was reduced. Once the antibiotic was removed, the HR clones generated were tested for the virD gene by PCR to confirm that they were free of agrobacterium infection (Fig. 2). In general, the presence of the transgenes did not affect root morphology, which was very similar to the wild type HR. Several root clones with a high growth capacity (growth index, GI > 3.5) and displaying the typical HR phenotype were selected, and the presence of the respective transgenes in their genome was further confirmed by PCR (Fig. 2). Remarkably, only 23% of roots generated after co-infection with 3 *Agrobacterium* reached the aforementioned GI, probably due to the presence of both antibiotics in the culture medium, in contrast with 37% of those cultivated only with kanamycin. The verified presence of the VvSTS gene, AtMYB12 TF and the amiRNA-CHS in the selected root lines allowed us to establish four tobacco HR populations (Table 1) for further experiments.

**Table 1:** Description of the different hairy root lines established, indicating the plasmids used for agroinfection and the transgenes integrated in the plant genome and confirmed by PCR.

Line type	Plasmids	Transgenes inserted
C	pRiA4	T-DNA
S	pRiA4 + pJCV52-STS3	T-DNA + VvSTS
TF/S	pRiA4 + pJCV52-STS3, pRiA4 + pJCV52-AtMYB12	T-DNA + VvSTS + AtMB12 TF
TF/S/Inh	pRiA4 + pJCV52-STS3, pRiA4 + pJCV52-AtMYB12, pRiA4 + pDMDC32B-AtMiR390a-B/c-amiRNACHS	T-DNA + VvSTS + AtMB12 TF + amiRNA-CHS

### Transcriptional analysis of the introduced transgenes and their effects on the expression of phenylalanine ammonia lyase and chalcone synthase and the production of stilbenes, total phenolics and total flavonoids

**Figure 3:**
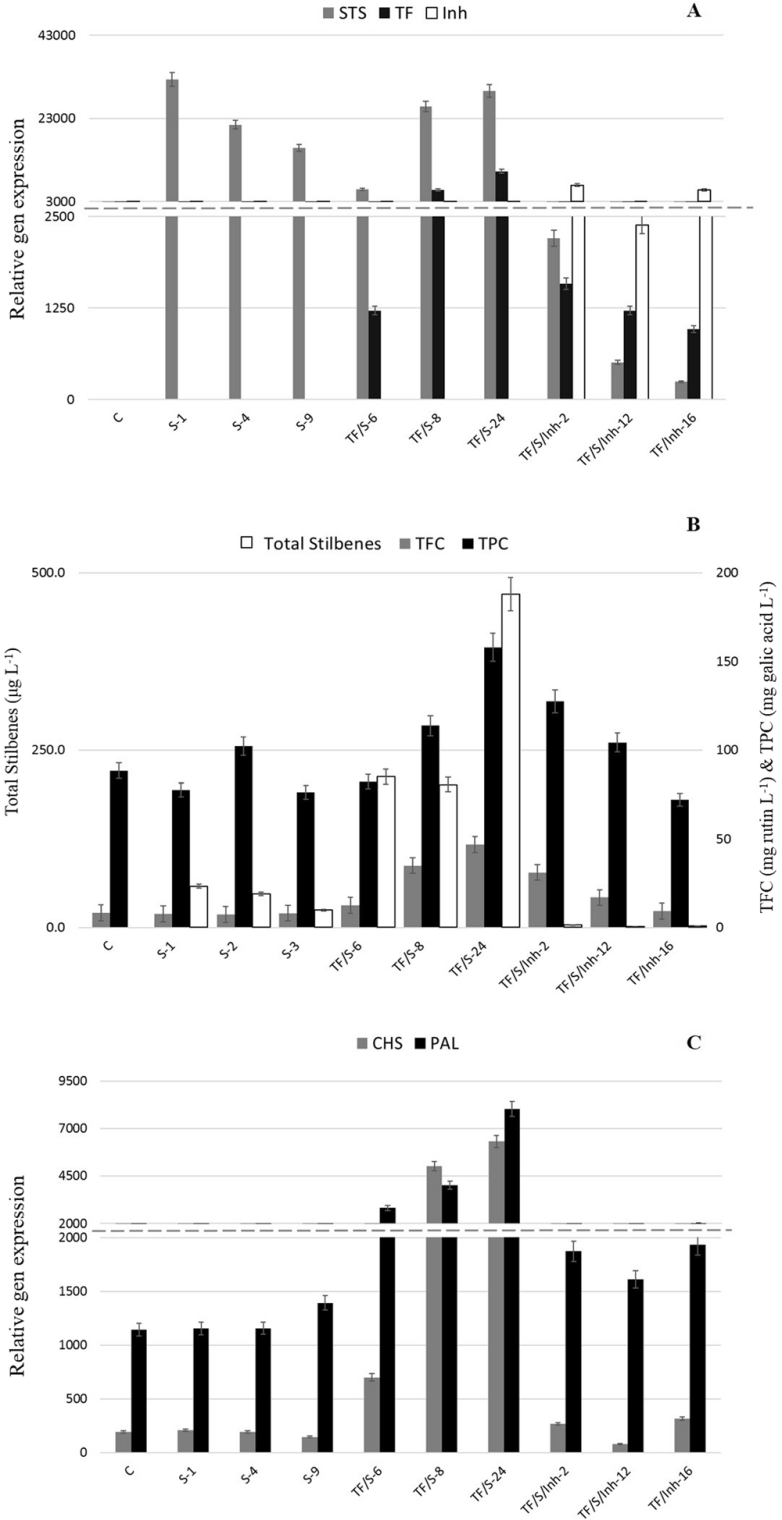
(**A**) Relative gene expression of STS, stilbene synthase; Inh, amiRNA-CHS and TF, AtMYB12 TF. (**B**) Total stilbene content measured as the sum of *t*-R, *t*-Pn, *t*-Pt and piceid expressed as μg/L; TFC, total flavonoid content expressed as rutin (mg/L); TPC, total phenolic content expressed as GA, gallic acid (mg/L). (**C**) Relative gene expression of CHS, chalcone synthase, and PAL, phenylalanine ammonia lyase. Each value is the average of 6 biological replicates ± SE.

Ten selected HR clones of the different types (denoted as C, S-1, S-4, S-9; TF/S-6, TF/S-8, TF/S-24 and S/TF/Inh-2, TF/S/Inh-12, TF/S/Inh-16) were transferred to a liquid medium and subcultured every two weeks. After several rounds of subculture, samples were taken in sextuplicate for each analysis. The qRT-PCR experiments revealed transgene expression driven by the CaMV 35 S promotor in all the selected HR lines, although at variable levels (Fig. 3A). The expression of the VvSTS gene was notably low in the S/TF/Inh transgenic roots, even though we took into account the identity between the nucleotide sequence of endogenous CHS and VvSTS (70%) and verified a non-homologous zone to select the amiRNA (see Supporting Information). In contrast, as expected, no interactions between AtMB12 TF and VvSTS expression were observed, because both genes were under the control of the 35S promotor (Fig. 3A).

Among the metabolic changes induced by the heterologous expression of the different transgenes, stilbene biosynthesis was driven by the VvSTS gene, resulting in 57.4 μg L^−1^ of total stilbenes (*t*-R + *t*-Pt + *t*-Pn + Piceid), which were not detected in the wild type HR (C lines; Fig. 3B). These results demonstrate that the designed biotechnological platform carrying an active STS enzyme was able to produce *t*-R and then metabolize it to other stilbenes by the action of unspecific tobacco enzymes constitutively present in the HR cultures. As shown in Fig. 3B, Table S2, the VvSTS gene expression was significantly correlated with the total stilbene content in the HR clones.

As expected, the co-expression of the AtMYB12 TF-boosted stilbene biosynthesis and the total stilbene content in TF/S-type HR lines was more than 6.9-fold higher than in the S-type. A similar increase in total phenolic compounds (TPC) (3.9-fold) and total flavonoids (TFC) (2.3-fold) also occurred in the TF/S-type roots (Fig. 3B). These results show that the TF effectively enhanced phenolic metabolism in the tobacco HR cultures (Fig. 1). Furthermore, there was a significant positive correlation between the transcript levels of the AtMYB12 TF and those of the phenylalanine ammonia-lyase (PAL) (ccP = 0.970) and CHS (ccP = 0.967) genes from tobacco (Figs 1 and 3C), indicating that the TF could activate early and late genes involved in phenolic metabolism in the tobacco HR cultures.

On the other hand, decreased levels of TFC (Fig. 3B) were observed in the HR lines carrying the amiRNA-CHS (TF/S/Inh lines), confirming an interference with the natural CHS gene expression in the biotechnological system (Fig. 3C). This was also ratified by the negative correlation between the amiRNA and CHS gene expression (ccP = −0.665) (Fig. 1).

### The metabolomic perturbations induced by the AtMYB12 TF

**Figure 4:**
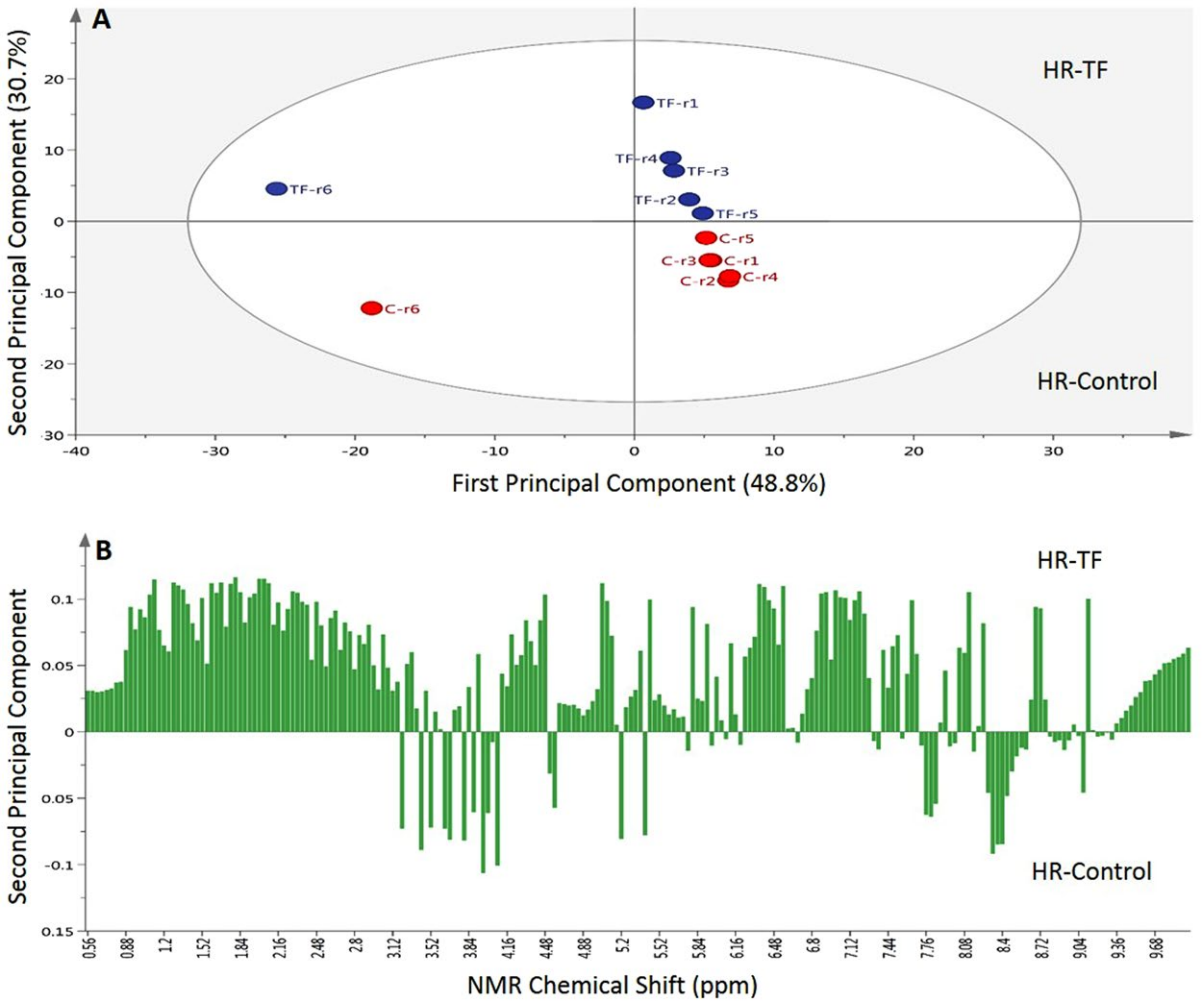
Score plot of principal component analysis (PCA) obtained from all ^1^H NMR data using PC1 and PC2 (**A**) and the corresponding loading column plots (**B**) of control hairy roots (HR) and transgenic HR, harbouring the AtMYB12 TF. Results are the average of 6 biological replicates.

In order to examine the metabolomic alterations caused by the expression of the AtMYB12 TF, excluding those induced by the VvSTS gene or the amiRNA-CHS, we obtained transgenic HR clones carrying the T-DNA of *A. rhizogenes* together with the AtMYB12 TF. The presence of the transgene was confirmed by PCR (see Fig. S2). ^1^H-NMR fingerprinting coupled with principal component analysis (PCA) allowed the identification of major perturbations in the metabolome of the transgenic roots harboring the AtMYB12 TF in comparison with wild type HR (control line). To examine in depth the metabolomic changes, the large data set from the ^1^H-NMR was subjected to PCA to reduce the numerous NMR signals of the extracts (Fig. 4). A total of 79.5% of model variance was described with the principal components PC1 and PC2 (Fig. 4A), revealing significant metabolomic differences. Both groups of samples were clearly divided by component two (Fig. 4A) and the amino acid (δ 0.5–3.0 ppm), carbohydrate (δ 3.0–5.5 ppm) and aromatic (δ 5.5–9.8 ppm) regions were highly clustered in the root line with the AtMYB12 TF (Fig. 4B). The signals of compounds detected by NMR are included in the Table S3.

**Figure 5:**
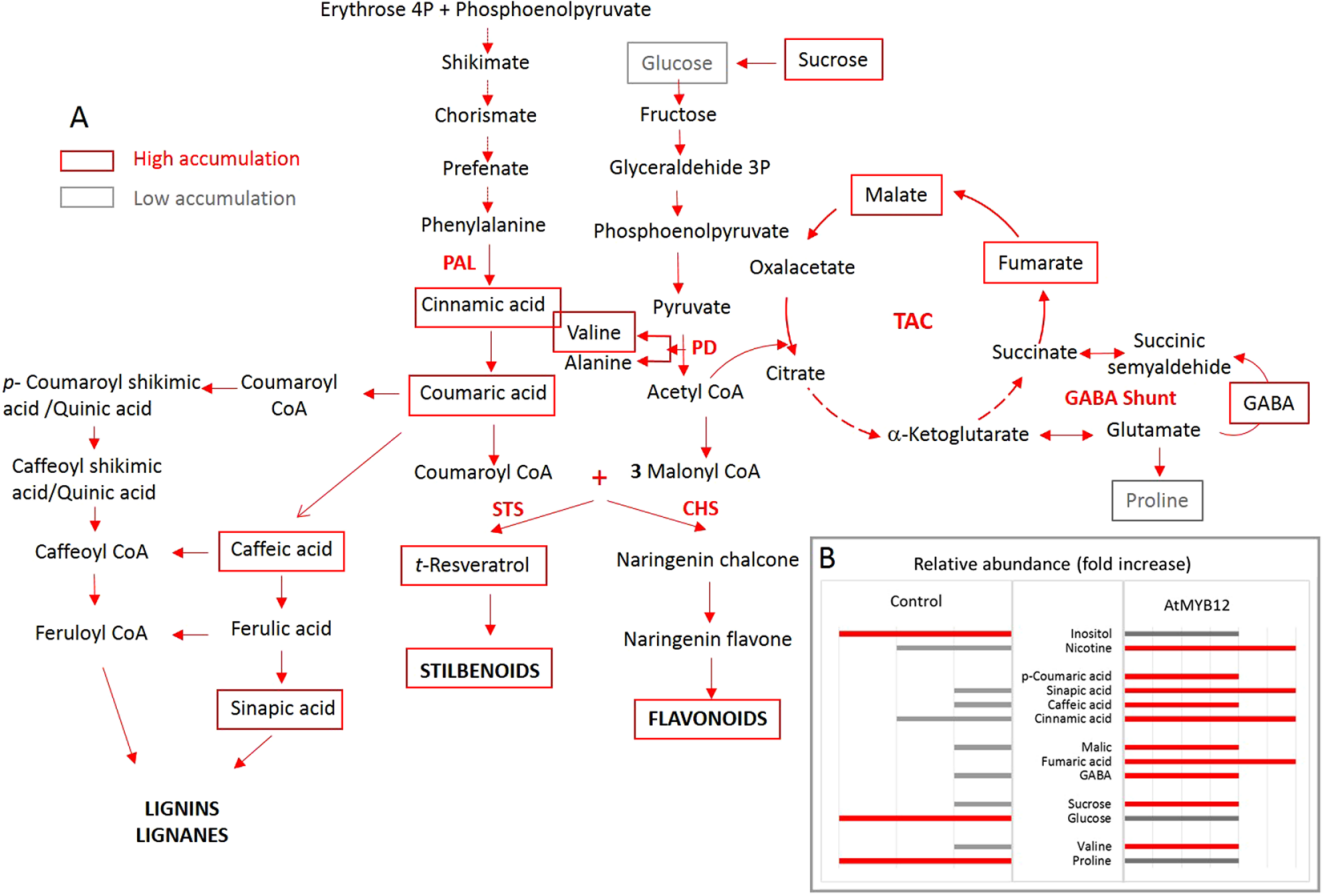
(**A**) Proposed metabolomic alterations in the tobacco HR lines due to the expression of the AtMYB12 TF. Red boxes, increased pathway/compounds. Grey boxes, decreased pathway/compounds. (**B**) Relative abundance (fold increase) of compounds in the transgenic AtMYB12 TF root lines compared with the wild type root lines (in red, increased compounds, in grey, decreased compounds).

The TF significantly affected the organic acid profile, both qualitatively and quantitatively, especially that of phenolic acids. The accumulation of two acids from the tricarboxylic acid cycle (TAC), fumaric and malic acid, as well as GABA, was notably enhanced (Fig. 5A,B, Table S3). Also, together with higher levels of cinnamic and *p*-coumaric acid, which are involved in the biosynthesis of stilbenes and flavonoids, the transgenic roots accumulated increased levels of caffeic and sinapic acid, which are eventually involved in lignin/lignan biosynthesis (Fig. 5A,B, Table S3). Similar glutamine, leucine or threonine levels were observed in both transgenic and wild type HR; in contrast, the contents of proline and valine were significantly affected by the expression of AtMYB12 TF (Fig. 5A,B, Table S3).

### Bioproduction of stilbenes in elicited hairy roots

**Figure 6:**
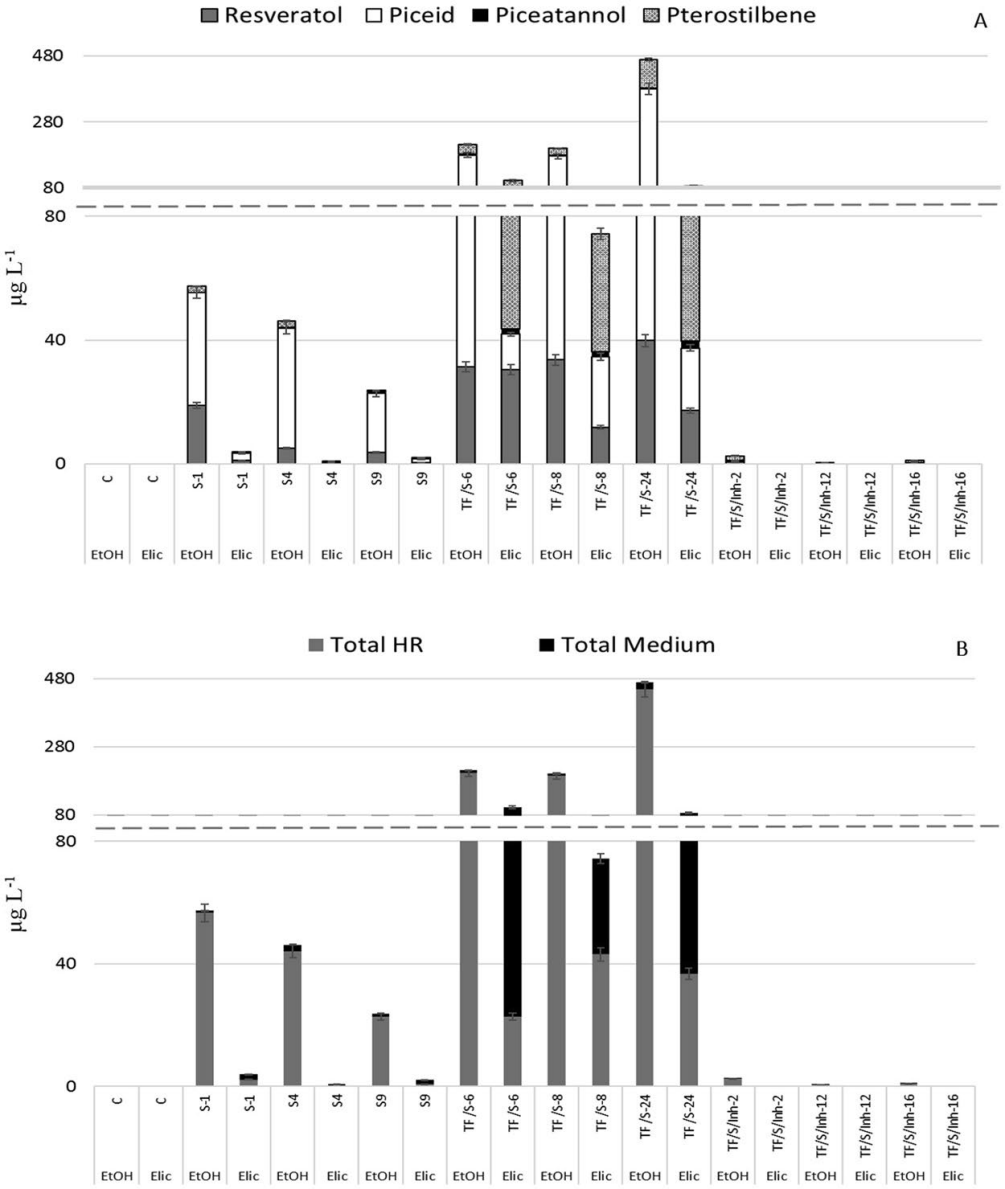
Stilbene production expressed as μg/L in the different HR clones studied after a 2-week culture period. (**A**) Stilbene pattern accumulation. (**B**) Stilbene distribution in the HR or culture medium. Elic, elicited (MBCD + MeJA) conditions; EtOH, control conditions. Each value is the average of 6 biological replicates ± SE.

In order to investigate the effects of elicitation on the stilbene production in the designed biotechnological system, 100 μM methyl jasmonate (MeJA) and 50 mM methylated β-cyclodextrin (MBCD) were added to the tobacco HR cultures after 11 days of culture and samples were taken 3 days later. This elicitor treatment has been reported as optimum for inducing *t*-R production in *V. vinifera* cell cultures^16^. Under the non-elicited control conditions, total stilbene accumulation was highest in roots harboring both the VvSTS gene and AtMYB12 TF (TF/S lines). Specifically, the root line TF/S-24, which displayed the highest expression of both transgenes (Fig. 3A), reached a stilbene production of 468.6 μg L^−1^ (Fig. 6A). In most lines, the stilbene accumulation pattern was piceid > *t*-R > *t*-Pt > *t*-Pn. In the TF/S-type lines, the contents were 142.4–339.7 μg L^−1^ for piceid, and 31.3–39.9 μg L^−1^ for *t*-R; the maximum yield of *t*-Pt was 86.4 μg L^−1^, whereas *t*-Pn levels were significantly lower at 2.2–3.4 μg L^−1^ (Fig. 6A). These results show that unspecific tobacco methyltransferases effectively bioconverted *t*-R into *t*-Pt when the AtMYB12 TF was co-expressed with STS in the root cultures. On the other hand, the lowest stilbene production occurred in the TF/S/Inh-type root lines (Fig. 6A), which may be related with the concomitant low expression of the VvSTS and AtMYB12 TF genes (Fig. 3A).

The combined elicitor treatment (MeJA + MBCD) did not increase total stilbene production in the studied transgenic HR (Fig. 6A), but it profitably changed the stilbene pattern to t-Pt > *t*-R ≥ piceid > *t*-Pn, with a notable increase in the two most active derivatives, *t*-Pt and *t*-Pn, whose yields were on average 200% and 80% higher, respectively. However, as shown in Fig. 6B, the elicitor treatment was extremely effective in releasing the accumulated stilbenes into the culture medium, with only piceid remaining in the root tissues.

## Discussion

As mentioned, tobacco is a useful model plant system, which can be easily transformed by *A. rhizogenes* to generate HR cultures and this trait may be harnessed for the heterologous expression of foreign genes harbored in engineered *A. rhizogenes* strains^43^. This fact, along with the growing interest in *t*-R and its derivatives *t*-Pn and *t*-Pt^14,15^, prompted us to set up a production platform based on transgenic tobacco HR. Thus, together with the *A. rhizogenes* T-DNA, the engineered tobacco HR lines harbored the following: the VvSTS gene for heterologous *t*-R production; AtMYB12 TF from *Arabidopsis thaliana* together with VvSTS to generate a general response in the phenylpropanoid pathway and coordinate the up-regulation of multiple steps^35^; and amiRNA-CHS together with STS and AtMYB12 TF to limit the phenolic flux through the endogenous CHS enzyme, which competes for precursors with the STS enzyme imported for the flux redirection.

Our results confirmed the potential of engineered HR cultures for *t*-R production, and the natural capacity of tobacco roots to bioconvert *t*-R into derivatives such as piceid, *t*-Pn and *t*-Pt. The bioproduction of *t*-R was apparently strongly correlated with the expression level of the VvSTS gene in the different transgenic HR clones obtained.

The genetic transformation of plants with STS genes has given interesting results, including plant disease resistance^44,45^, and is still being intensively studied^8^. In this research pipeline, the first experiments were performed by Jeandet *et al*.^46^, who expressed the STS gene from *Arachis hypogea* in tobacco plants, leading to resveratrol accumulation after treatment with short-wavelength ultraviolet light. Heterologous expression of the STS gene has subsequently been reported in several edible plant species, including tomato, kiwifruit, apple, lettuce, barley and wheat, as a defense against plague attack, based on the phytoalexin activity of resveratrol^8^.

Several metabolic engineering strategies have also been developed for *t*-R production in microorganisms, but unlike plant systems, these require the introduction of a complex set of genes. In this context, the entire resveratrol pathway has been induced in modified yeast, *Yarrowia lipolytica* (ATCC 20362 strain), including the genes encoding phenylalanine/tyrosine ammonia lyase (PAL/TAL), cinnamate-4-hydroxylase (4CH), *p*-coumaroyl-CoA ligase (4CL), and STS^47^. More recently, Trantas *et al*.^48^ constructed the complete resveratrol biosynthetic pathway in *S. cerevisiae* to produce resveratrol from the precursor phenylalanine (10 mM), obtaining a content of 290 μg L^−1^ *t*-R after 120 h of culture. Higher yields have been achieved in engineered *Escherichia coli* strains; for instance, Katsuyama *et al*.^49^ reported a production of 171 mg L^−1^ of *t*-R. However, the production of *t*-R in microorganisms always entails the addition of exogenous precursors^50^.

The production of *t*-Pn and *t*-Pt was recently reported by Li *et al*.^51^ in engineered yeast after feeding the culture with phenylalanine, and Wang *et al*.^52^ obtained *t*-Pt from *t*-R and *p*-coumaric acid in both engineered yeast and *E. coli*. In seed plant systems, the production of *t*-Pn and *t*-Pt has been enabled by engineering either *V. vinifera* plant cell cultures^14^ or tobacco transgenic HR^15^. In both cases the heterologous genes HsCYP1B1 and VvROMT were constitutively expressed, whilst *t*-R was induced by elicitation in the cell cultures or supplied by direct feeding in the HR.

As mentioned in the introduction, TF overexpression can boost a complete metabolic pathway^3^. In this context, the TF of the MYB family have been described as the most useful in enhancing flavonoid biosynthesis^31,32^. In tobacco plants, AtMYB12 TF expression causes a strong transcriptome change by up-regulating genes encoding enzymes downstream in the phenolic pathway, which increases flavonoid content, as well as the expression of several genes encoding upstream enzymes involved in the biosynthesis of common precursors in the stilbene and flavonoid biosynthetic pathways^34^.

In our study, in agreement with the results reported by Misra *et al*.^34^, AtMYB12 TF expression was tightly correlated with an enhanced flavonoid content in the transgenic HR clones, probably because of an up-regulation of the CHS gene. It has previously been demonstrated that ectopic expression of the AtMYB12 TF increases the flavonoid content of tomato leaves and fruits^33,35^. In our tobacco HR, the TF also enhanced the expression of the upstream PAL, resulting in a higher total phenolic accumulation compared with the control roots. Similar results have been reported in AtMYB12 TF transgenic tobacco plants, although PAL expression was not regulated by the TF in *Arabidopsis thaliana* ^32^.

A more detailed view of the metabolism modulation caused by ectopic expression of MYB12 TF in transgenic HR was provided by an NMR-based metabolomics approach. Major qualitative and quantitative metabolomic changes were analyzed in roots expressing only the AtMYB12 TF and compared with wild type HR (control line). As well as amino acids and sugars, the transgene expression affected organic acids, especially phenolic acids. The accumulation of fumaric and malic acids from the tricarboxylic acid cycle was notably enhanced, and high levels of GABA were found, but contrary to previous studies of transgenic plants, citrate contents were not affected^34^. These results reflect that the regulation of metabolic pathways varies according to the plant organs and tissues. Thus, while the levels of several amino acids, including valine, alanine, and phenylalanine, increased in the MYB-12 transgenic tobacco plants, in the HR system we observed the up-regulation only of valine, and the down-regulation of proline biosynthesis.

Among phenolic acids, the transgenic roots accumulated higher levels of cinnamic and *p*-coumaric acid, which are involved in the biosynthesis of stilbenes and flavonoids, as well as caffeic and sinapic acid, which may be involved in lignin biosynthesis. These results are in agreement with those reported for AtMYB12 TF transgenic tobacco plants by Misra *et al*.^34^, who observed an increased expression of genes involved in lignin biosynthesis. Altogether, the results suggest that part of the increased precursor availability for stilbene biosynthesis could be redirected to the lignin pathway, undermining the effectiveness of our biotechnological system for the production of the target compounds, *t*-R and its derivatives *t*-Pn and *t*-Pt (Fig. 5).

The heterologous expression of STS can alter flower morphology, and cause male sterility in tobacco and petunia^53^. The sterility may be linked to a competition for substrates between STS and endogenous CHS, since fertility can be restored in tobacco by adding exogenous flavonol^53^. As mentioned, STS and CHS are both involved in the polyphenol pathway, and it is reasonable to assume that the expression of exogenous STS may lead to a competition for substrates. Indeed, among the analyzed tobacco HR lines, those transformed with amiRNA-CHS achieved the lowest levels of flavonoids due to inhibition of the CHS gene expression. By silencing the branching point of the flavonoid biosynthetic pathway, a greater flow of precursors was channeled toward the production of *t*-R and its derivatives. Unfortunately, the transgenic root lines carrying the amiRNA-CHS also showed a low VvSTS gene expression and their stilbene contents were lower than in the TF/S root clones. Differences in transgene expression among HR clones can be attributed to transgene rearrangements, which may reduce their expression^54^. It is known that exogenous DNA sequences can undergo recombination, rearrangement and truncation before or during integration but not afterwards^55^, which could have a significant effect on transgene expression. Random transgene insertion into the plant genome is another possible reason for the differences in transgene expression among HR lines^56,57^.

We have previously discussed that elicitation can improve the heterologous production of a target compound in plant cell cultures^58,59^. Elicitation of the engineered HR with 50 mM MBCD and 100 µM of MeJA significantly increased the release of *t*-R and its derivatives *t*-Pn and *t*-Pt to the liquid medium, whereas piceid remained mainly inside the roots. In contrast, the positive effects of the combined elicitor treatment (MBCD + MeJA) on the *t*-R production has been previously reported in non-transgenic and transgenic grapevine cell cultures^14,60^, and also in transgenic *S. marianum* cell cultures carrying the VvSTS gene and supplemented with MBCD^17^. The ineffectiveness of elicitation in the HR culture system to improve the total stilbenoid contents was perhaps due to the fast metabolization of stilbenoids in the culture medium, as reported previously^15^, and an increased bioconversion of *t*-R into the more bioactive derivatives, *t*-Pn and *t*-Pt.

## Conclusions

Taken as a whole, our results show the suitability of the engineered HR cultures for the heterologous production of *t*-R and its derivatives *t*-Pn and *t*-Pt. The effectiveness of the AtMYB12 TF for activating the phenolic pathway and amiRNA CHS for blocking competitive pathways was also demonstrated. However, these results also show the extreme complexity of metabolically engineering biotechnological systems based on *in vitro* seed plants. A large part of the *t*-R biosynthesized by the root cultures was metabolized not only to the target compounds *t*-Pn and *t*-Pt, but also to piceid and probably other non-identified resveratrol derivatives. Moreover, the boosting of phenolic metabolism, reflected by the high levels of caffeic and sinapic acid in the HR lines harboring the AtMYB12 TF, could also provide precursors for competitive pathways, such as lignan and lignin biosynthesis. Therefore, new approaches are required in which competitive upstream pathways are blocked by specific amiRNAs and the carbon flux is more effectively channeled to stilbene biosynthesis.

## Methods

### Bacteria and plasmids

Four strains of *Agrobacterium rhizogenes* A4 were used: wild type and three engineered strains carrying the pRiA4. The specific engineered strains and binary plant expression vectors of each one are described in Table 2 and Fig. S1.

**Table 2:** Transgenic *Agrobacterium rhizogenes* A4 utilized in the experiments.

*A. rhizogenes* ID	Expression vector/Gene	Antibiotic for HR selection
A4-S	pJCV52/Stilbene synthase 3 (VvSTS-3)	Kanamycin
A4-TF	pJCV52/*Arabidopsis thaliana* transcription factor AtMYB12	Kanamycin
A4-Inh	pMDC32B - AtMIR390a-B/c/Artificial microRNA of CHS (amiRNA-CHS) into *Arabidopsis thaliana* MIR390a precursor	Hygromycin

The VvSTS gene (Acc. XM_002264953) was cloned as described by Martinez-Marquez *et al*.^14^. The clone TOPO-U04-A02 that contains the AtMYB12 gene was purchased from the *Arabidopsis* Biological Resource Center (ABRC). An LR recombination reaction was performed to generate an expression clone using LR Clonase™ (Invitrogen, Carlsbad, CA). The construction of amiRNA-CHS (see Supp information) was as described by Carbonell *et al*.^42^ using the following sequences: forward oligo: 5′TGT ATT AAT CAT TGA TTT TTC ACA GAT GAT GAT CAC ATT CGT TAT CTA TTT TTT CTG TGA AAA AGC AAT GAT TAA 3′; reverse oligo: 5′ATG TTA ATC ATT GCT TTT TCA CAG AAA AAA TAG ATA ACG AAT GTG ATC ATC ATC TGT GAA AAA TCA ATG ATT AA 3′. The *Agrobacterium* strains were transformed by electroporation as described by Shaw^61^.

### Stable transformation and hairy root culture induction

Leaf discs of *Nicotiana tabacum* cv Xhanti plantlets grown *in vitro* on Murashige and Skoog (MS) medium were co-infected as described by Gallois and Marinho^62^, using a wild type *A. rhizogenes* A4 or A4-S or A4-S plus A4-TF or A4-S plus A4-TF plus A4-Inh. After 2–4 weeks, HR appeared in leaf discs maintained on MS solid medium with 30 g L^−1^ of sucrose and 500 mg L^−1^ cefotaxime to eliminate the agrobacteria. Further, HR were excised and individually cultured on the same medium plus kanamycin (50 mg L^−1^) and/or hygromycin (50 mg L^−1^) for selection, depending on the target genes (i.e. HR obtained by co-infection with 3 *Agrobacterium* was cultivated for selection with kanamycin and hygromycin). The tobacco HR were kept in these conditions at 25 °C in the dark for several rounds of subculturing until the elimination of agrobacteria and confirmation by PCR analysis.

### PCR analysis

The analysis was performed with DreamTaq Green PCR Master Mix (Thermo Fisher Scientific Inc). Previously, genomic DNA was isolated as described by Dellaporta *et al*.^63^. The specific primers and amplification reactions used are described in Table S1. PCR products were analyzed by electrophoresis on 1% agarose gels.

### Gene expression by qPCR analysis

Expression of th*e* VvSTS, AtMYB12, amiRNA-CHS, PAL and CHS genes was verified by qPCR in selected HR lines as described by Hidalgo *et al*.^15^. Briefly, total RNA was isolated with TRIzol reagent (Invitrogen, Carlsbad, CA) and treated with DNase I (Invitrogen, Carlsbad, CA). Then, cDNA was synthesized with SuperScript III reverse transcriptase (Invitrogen, Carlsbad, CA) and finally, qRT-PCR was performed with iTAqTM Universal SYBR Green Supermix (BioRad, Hercules, CA, EEUU) in a 384-well platform system (LightCycler 480 Instrument; Roche). Conditions and primers used are described in Table S1.

### Stilbene extraction and determination

The HR and culture medium were processed as described by Hidalgo *et al*.^15^. Briefly, two times one volume of ethyl acetate was added per four volumes of the medium, stirring vigorously, and the non-polar solvent recovered and evaporated. For the HR, 50 mg of freeze-dried powder was extracted twice with 2 volumes of 100% methanol, sonicated for 30 min, and the supernatant was collected and evaporated. Stilbenes were determined by a Linear Ion Trap Quadrupole LC/MS/MS Mass Spectrometer, 4000 Q TRAP of AB Sciex Instruments with MRM scan type in negative mode as described by Hidalgo *et al*.^15^.

### Total phenolic content (TPC)

The compounds were extracted as indicated in the previous paragraph and their quantity was estimated by the Folin-Ciocalteu colorimetric method as described by Thiruvengadam *et al*.^64^ with slight modifications. Briefly, 100 µL of HR extracts were mixed with 3.1 mL of distilled water, followed by addition of 0.2 mL Folin-Ciocalteu reagent. After 5 min, 0.6 mL of 20% sodium carbonate solution was added, and after 60 min of incubation the solution absorbance was measured at 760 nm. The concentration was calculated as mg of gallic acid equivalents using a calibration curve.

### Total flavonoid content (TFC)

The flavonoid quantity in extracts was estimated using the aluminum chloride colorimetry method described by Thiruvengadam *et al*.^64^ with slight modifications. Briefly, 200 µL of extract, 100 µL of 10% (w/v) aluminum chloride solution, 100 µL of 1 M potassium acetate solution, and 4.6 mL of distilled water were mixed. After 30 min of incubation the solution absorbance was measured at 415 nm. The concentration was calculated as mg of rutin equivalents using a calibration curve.

### Sample preparation, NMR conditions and data analysis

Six biological replicates of HR were treated as described by Zahmanov *et al*.^65^. Briefly, in a 2 mL tube 50 mg freeze-dried HR powder, 750 µL of CD_3_OD and 750 µL of D_2_O (KH_2_PO_4_, buffer, pH 6.0, containing 0.01% w/v TSPA-d4) were mixed. The mixture was homogenized, sonicated for 20 min, and centrifuged for 20 min at 12,000 rpm. Finally, 800 µL was placed into the NMR tube. The proton spectra (^1^H NMR) were recorded at 25 °C on an AVII+ 600 spectrometer (Bruker, Karlsruhe, Germany) at a proton frequency of 600.13 MHz with 4.07 s relaxation time and CD_3_OD as the internal lock^66^. The data analysis was processed as described by Marchev *et al*.^67^. Briefly, the spectra were phased, baseline corrected, set to TSPA at 0.0 ppm and binned to 0.04 ppm using MestReNova software (version 6.2.1, Mestrelab Research, Santiago de Compostela, Spain). The principal component analysis (PCA) was performed with SIMCA-P14.0 (Umetrics, Umea, Sweden), excluding the signals of water and methanol.

### Elicitation assays

The selected HR clones were cultivated in a 200 mL flask with 0.5 g per 25 mL of liquid MS medium at 115 rpm, 25 °C in darkness. After 11 days, MeJA and MBCD at final concentrations of 100 µM and 50 mM, respectively, were added. After 3 days of elicitation, samples were taken with their respective control conditions.

### Statistics

The statistical analysis was performed with Microsoft Excel software. All data are the average of three measurements + SE. The multifactorial ANOVA analysis followed by the Tukey multiple comparison test were used for statistical comparisons. A p-value of < 0.05 was assumed for significant differences. Correlation studies (Pearson’s correlation) were performed considering the p value < 0.1 as significant.

## Electronic supplementary material

Supplementary material

## References

[1] Baur JA, Sinclair DA (2006). Therapeutic potential of resveratrol: the *in vivo* evidence. Nat. Rev. Drug Discov..

[2] Piotrowska H, Kucinska M, Murias M (2012). Biological activity of piceatannol: leaving the shadow of resveratrol. Mutation Res..

[3] Szekeres T, Saiko P, Fritzer-Szekeres M, Djavan B, Jäger W (2011). Chemopreventive effects of resveratrol and derivatives. Annals N.Y. Acad. Sci..

[4] McCormack D, McFadden D (2013). A review of pterostilbene antioxidant activity and disease modification. Oxid. Med. Cell Longev..

[5] Srivastava, V., Mehrotra, S. & Mishra, S. Biotransformation through hairy roots: Perspectives, outcomes, and major challenges. In: *Transgenesis and Secondary Metabolism* (ed. Jha, S.) 1–24. (Springer International Publishing 2016).

[6] Georgiev MI, Weber J, MacIuk A (2009). Bioprocessing of plant cell cultures for mass production of targeted compounds. Appl. Microbiol. Biotechnol..

[7] Georgiev MI, Agostini E, Ludwig-Mueller J, Xu J (2012). Genetically transformed roots: from plant disease to biotechnological resource. Trends Biotechnol..

[8] Kim Y, Wyslouzil BE, Weathers PJ (2002). Secondary metabolism of hairy root cultures in bioreactors. In Vitro Cell. Dev. Biol. – Plant..

[9] Sevón N, Oksman-Caldentey KM (2002). *Agrobacterium rhizogenes*-mediated transformation: Root cultures as a source of alkaloids. Planta Med..

[10] Chandra S, Chandra R (2011). Engineering secondary metabolite production in hairy roots. Phytochem. Rev..

[11] Rischer H (2013). Plant cells as pharmaceutical factories. Curr. Pharm. Des..

[12] Delaunois B, Cordelier S, Conreux A, Clément C, Jeandet P (2009). Molecular engineering of resveratrol in plants. Plant Biotechnol. J..

[13] Kiselev KV (2011). Perspectives for production and application of resveratrol. Appl Microbiol Biotechnol..

[14] Martínez-Márquez A (2016). Production of highly bioactive resveratrol analogues pterostilbene and piceatannol in metabolically engineered grapevine cell cultures. Plant Biotechnol. J..

[15] Hidalgo D (2017). Bioconversion of stilbenes in genetically engineered root and cell cultures of tobacco. Sci. Rep..

[16] Lijavetzky D (2008). Synergistic effect of methyljasmonate and cyclodextrin on stilbene biosynthesis pathway gene expression and resveratrol production in Monastrell grapevine cell cultures. BMC Res. Not..

[17] Hidalgo D (2017). *Silybum marianum* cell cultures stably transformed with *Vitis vinifera* stilbene synthase accumulate *t*-resveratrol in the extracellular medium after elicitation with methyl jasmonate or methylated β-cyclodextrins. Eng. Life Sci..

[18] Yang T (2015). Enhanced production of resveratrol, piceatannol, arachidin-1, and arachidin-3 in hairy root cultures of peanut co-treated with methyl jasmonate and cyclodextrin. J. Agric. Food Chem..

[19] Halder M, Jha S (2016). Enhanced trans-resveratrol production in genetically transformed root cultures of Peanut (*Arachis hypogaea* L.). Plant Cell. Tiss. Organ Cult..

[20] Nopo-Olazabal C, Condori J, Nopo-Olazabal L, Medina-Bolivar F (2014). Differential induction of antioxidant stilbenoids in hairy roots of *Vitis rotundifolia* treated with methyl jasmonate and hydrogen peroxide. Plant Physiol. Biochem..

[21] Capell T, Bassie L, Christou P (2004). Modulation of the polyamine biosynthetic pathway in transgenic rice confers tolerance to drought stress. Proc. Natl. Acad. Sci. USA.

[22] Capell T, Christou P (2004). Progress in plant metabolic engineering. Curr. Opin. Biotechnol..

[23] Naqvi S (2010). When more is better: multigene engineering in plants. Trends Plant Sci..

[24] Zhu C (2008). Combinatorial genetic transformation generates a library of metabolic phenotypes for the carotenoid pathway in maize. Proc. Natl. Acad. Sci. USA.

[25] Farré G (2014). Engineering complex metabolic pathways in plants. Ann. Rev. Plant Biol..

[26] Fukushima A, Kusano M, Redestig H, Arita M, Saito K (2009). Integrated omics approaches in plant systems biology. Curr. Opin. Chem. Biol..

[27] Jacobs DI, Van Der Heijden R, Verpoorte R (2000). Proteomics in plant biotechnology and secondary metabolism research. Phytochem. Anal..

[28] Wang H, Wang H, Shao H, Tang X (2016). Recent advances in utilizing transcription factors to improve plant abiotic stress tolerance by transgenic technology. Front. Plant Sci..

[29] Wilson SA, Roberts SC (2012). Recent advances towards development and commercialization of plant cell culture processes for the synthesis of biomolecules. Plant Biotechnol. J..

[30] Christou, P. & Klee, H. Handbook of plant biotechnology. John Wiley & Sons (2004).

[31] Mehrtens F, Kranz H, Bednarek P, Weisshaar B (2005). The *Arabidopsis* transcription factor MYB12 is a flavonol-specific regulator of phenylpropanoid biosynthesis. Plant Physiol..

[32] Stracke R (2007). Differential regulation of closely related R2R3-MYB transcription factors controls flavonol accumulation in different parts of the *Arabidopsis thaliana* seedling. Plant J..

[33] Luo J (2008). AtMYB12 regulates caffeoyl quinic acid and flavonol synthesis in tomato: Expression in fruit results in very high levels of both types of polyphenol. Plant J..

[34] Misra P (2010). Modulation of transcriptome and metabolome of tobacco by *Arabidopsis* transcription factor, AtMYB12, leads to insect resistance. Plant Physiol..

[35] Pandey A, Misra P, Choudhary D, Yadav R (2015). AtMYB12 expression in tomato leads to large scale differential modulation in transcriptome and flavonoid content in leaf and fruit tissues. Sci. Rep..

[36] Tiwari M, Sharma D, Prabodh, Trivedi K (2014). Artificial microRNA mediated gene silencing in plants: progress and perspectives. Plant Mol. Biol..

[37] Dodd AN (2005). Plant circadian clocks increase photosynthesis, growth, survival, and competitive advantage. Science..

[38] Kim J, Somers DE (2010). Rapid assessment of gene function in the circadian clock using artificial microRNA in *Arabidopsis* mesophyll protoplasts. Plant Physiol..

[39] Latijnhouwers M, Xu XM, Møller SG (2010). *Arabidopsis* stromal 70-kDa heat shock proteins are essential for chloroplast development. Planta.

[40] Schwartz C (2009). Cis-regulatory changes at Flowering Locus T mediate natural variation in flowering responses of *Arabidopsis thaliana*. Genetics.

[41] Toppino L (2011). Reversible male sterility in eggplant (*Solanum melongena* L.) by artificial microrna-mediated silencing of general transcription factor genes. Plant Biotechnol. J..

[42] Carbonell A (2014). New generation of artificial microRNA, synthetic trans-acting small interfering RNA vectors for efficient gene silencing in *Arabidopsis*. Plant Physiol..

[43] Vasilev N (2014). Comparison of plant-based expression platforms for the heterologous production of geraniol. Plant Cell Tiss. Organ Cult..

[44] Fischer R, Hain R (1994). Plant disease resistance resulting from the expression of foreign phytoalexins. Curr. Opin. Biotechnol..

[45] Hain, R. & Grimming, B. Modification of plant secondary metabolism by genetic engineering. In: *Metabolic Engineering of Plant Secondary Metabolism* (ed. Verpoorte, R., Alfermann, A.W.) 217–231 (Kluwer Academic Publishers 2000).

[46] Jeandet P (1997). HPLC analysis of grapevine phytoalexins coupling photodiode array detection and fluorometry. Anal. Chem..

[47] Huang, L. L., Xue, Z. & Zhu, Q. Q. Method for the production of resveratrol in a recombinant oleaginous microorganism. World Patent WO 2006125000 A2 (2006).

[48] Trantas E, Panopoulos N, Ververidis F (2009). Metabolic engineering of the complete pathway leading to heterologous biosynthesis of various flavonoids and stilbenoids in *Saccharomyces cerevisiae*. Metab. Eng..

[49] Katsuyama Y, Funa N, Miyahisa I, Horinouchi S (2007). Synthesis of unnatural flavonoids and stilbenes by exploiting the plant biosynthetic pathway in *Escherichia coli*. Chem. Biol..

[50] Jeandet P (2012). Metabolic engineering of yeast and plants for the production of the biologically active hydroxystilbene, resveratrol. J. Biomed. Biotechnol..

[51] Li M, Schneider K, Kristensen M, Borodina I, Nielsen J (2016). Engineering yeast for high-level production of stilbenoid antioxidants. Sci. Rep..

[52] Wang Y, Bhuiya MW, Zhou R, Yu O (2014). Pterostilbene production by microorganisms expressing resveratrol O-methyltransferase. Ann. Microbiol..

[53] Fischer R, Budde I, Hain R (1997). Stilbene synthase gene expression causes changes in flower colour and male sterility in tobacco. Plant J..

[54] Kohli A (2006). The quest to understand the basis and mechanisms that control expression of introduced transgenes in crop plants. Plant Signal Behav..

[55] Kohli A, Christou P (2008). Stable transgenes bear fruit. Nat Biotechnol..

[56] Butaye KMJ, Cammue BPA, Delauré SL, De Bolle MFC (2005). Approaches to minimize variation of transgene expression in plants. Mol. Breed..

[57] Hernandez-Garcia CM (2010). High level transgenic expression of soybean (*Glycine max*) GmERF and Gmubi gene promoters isolated by a novel promoter analysis pipeline. BMC Plant Biol..

[58] Exposito O (2010). Metabolic responses of *Taxus media* transformed cell cultures to the addition of methyl jasmonate. Biotechnol Prog..

[59] Mehrotra S, Rahman LU, Kukreja AK (2010). An extensive case study of hairy root cultures for enhanced secondary-metabolite production trough metabolic-pathway engineering. Biotechnol. Appl. Biochem..

[60] Jeandet P, Clement C, Courot E (2014). Resveratrol production at large scale using plant cell suspensions. Eng. Life Sci..

[61] Shaw, C. H. Introduction of Cloning Plasmids into *Agrobacterium tumefaciens*. In: Plant Gene Transfer and Expression Protocols. *Methods in Molecular Biology™* 49 (ed. Jones H.) 34–36 (Humana Press Inc. 1995). 10.1385/0-89603-321-X:33 8563816

[62] Gallois, P. & Marinho, P. Leaf Disk Tiansformation Using *Agrobacterium tumefaciens*-Expression of Heterologous Genes in Tobacco. In: Plant Gene Transfer and Expression Protocols. *Methods in Molecular Biology™* 49 (ed. Jones, H.) 41–46 (Humana Press Inc. 1995). 10.1385/0-89603-321-X:39 8563823

[63] Dellaporta SL, Wood J, Hicks JB (1983). A plant DNA minipreparation: Version II. Plant Mol. Biol. Rep..

[64] Thiruvengadam M (2014). Establishment of *Momordica charantia* hairy root cultures for the production of phenolic compounds and determination of their biological activities. Plant Cell Tiss. Organ Cult..

[65] Zahmanov G, Alipieva K, Simova S, Georgiev M (2015). Metabolic differentiations of dwarf elder by NMR-based metabolomics. Phytochem. Lett..

[66] Georgiev M (2015). Metabolic alterations of *Verbascum nigrum* L. plants and SAArT transformed roots as revealed by NMR-based metabolomics. Plant Cell Tiss. Organ Cult..

[67] Marchev AS, Aneva IY, Koycheva IK, Georgiev MI (2017). Phytochemical variations of *Rhodiola rosea* L. wild-grown in Bulgaria. Phytochem. Lett..

