# Supplementary material for: Tailoring tobacco hairy root metabolism for the production of stilbenes
Source: Sci Rep. 2017 Dec 21;7:17976. doi: 10.1038/s41598-017-18330-w (PMC5740106; doi:10.1038/s41598-017-18330-w)
Supplement: Supplementary file 1 — Supplementary material [file 41598_2017_18330_MOESM1_ESM.pdf]

Supplementary material:

Tailoring tobacco hairy root metabolism for the production of stilbenes

Diego Hidalgo, Milen Georgiev, Andrey Marchev, Roque Bru-Martínez, Rosa M. Cusidó, Purificación Corchete, Javier Palazon

Table S1. Sequences of the primers used to amplify the genes by PCR and qPCR

**PCR**			
Gene	Primer Sequence	Amplicon size bp	Reference
VvSTS	FW: ATGGCTTCAGTTGAGGAATTTAGA<br>RV: TTAATTTGTAACCGTAGGAACGCT	1179	Hidalgo et al. [1]
AtMYB12	FW: CACCATGGGAAGAGCGCCATGTTG<br>RV: TGACAGAAGCCAAGCGACCA	1117	This work
amiRNA-CHS	FW: ACGAAGAGGAGATGACGTGTG<br>RV: GTTTTGCCTCGGCCTTTTTCA	287	This work
rolC	FW: TAACATGGCTAGAAGACGACC<br>RV: AAACTTGCACTCGCCATGCC	534	Slightom, et al.[2]
virD	FW: ATGTCGCAAGGCAGTAAGCCCA<br>RV: GGAGTCTTTCAGCATGGAGCAA	438	Hirayama et al.[3]
Program: 95ºC for 5 min, 30 cycles (95ºC, 60 s; 60ºC, 40 s; 72ºC, 90 s) and an extension cycle of 10 min at 72ºC			
**qPCR**			
Elongation factor (qPCR)	FW: TGGTCAGGAGATTGCGAAAGAGC<br>RV: ACGCAAAACGCTCCAATGGTG	130	Hidalgo et al.[4]
STS (qPCR)	FW: AAGGGGAAAAAGCCACCACA<br>RV: TTCGATGGTCAAGCCAGGTC	77	Hidalgo et al.[1]
AtMYB12 (qPCR)	FW: GAGGCCACGATCGGGAATAG<br>RV: CAGAAGCCAAGCGACCAAAG	87	This work
amiRNA-CHS (qPCR)	FW: GAGATGACGTGTGTTCCTTCG<br>RV: GAGGAAGAAGGAGAAGAGAAGGTG	73	This work
PAL (qPCR)	FW: TCGAAGATGAATTGAAGGCTGTTTT<br>RV: GGAATAGCAGGGTTTCCACTTTCT	82	This work
CHS (qPCR)	FW: CAGCCGCGGTCATTATAGGT<br>RV: AGAGTTTGGGCTGCAGAGAC	82	This work
Program: 95°C for 2 min, 40 cycles (95°C, 10 s; 60°C, 20 s; 72°C, 20 s) followed by a melting curve			

Table S2. Analysis of the Pearson lineal correlation between the different parameters studied. ccP, Pearson correlation coefficient. TF, transcription factor: PAL, phenylalanine ammonia lyase; CHS, chalcone synthase, TFC, total flavonoids content; Stilb., total stilbenes content.

Related variable	ccP	p value
TF / PAL	0.970	0.001
TF / CHS	0.967	0.002
TF / TFC	0.882	0.020
TF / Stilb.	0.888	0.018
Inh / CHS	-0.665	0.072
Inh / TFC	-0.626	0.085
CHS / TFC	0.858	0.001
CHS / TPC	0.435	0.209
STS / Stilb	0.816	0.025
TF / TPC	0.423	0.403

Table S3. Metabolites identified by relevant spectra (1D and 2D NMR).

Group of compounds	Metabolite	Chemical shift (ppm)	Multiplicity/coupling constant (Hz)
Amino acids	Proline	4.08/2.31	(dd, *J* = 8.6; 6.0)/(m)
	Alanine	1.49	(d, *J* = 7.2)
	Glutamine	2.12/2.45	(m)/(m)
	Leucine	0.96/0.98	(d,*J* = 6.2)/(d, *J* = 6.4)
	Threonine	1.33	(d, *J* = 6.8)
	Valine	1.00/1.01	(d, *J* = 6.6)/(d, *J* = 7.1)
Sugar	α-Glucose	5.19	(d, *J* = 3.7)
	β-Glucose	4.58	(d, *J*= 7.9)
	Sucrose	5.41/4.17	(d, *J*=3.8)/(d,*J* = 8.6)
Organic acids	γ-Amino-butyrate (GABA)	1.9/2.32/3.01	(m)/(t,*J* = 7.5)/(t,*J* = 7.5)
	Acetic acid	1.94	(s)
	Fumaric acid	6.53	(s)
	Malic acid	2.82	(dd, *J* = 16.9; 8.1)
Phenolic acids	Cinnamic acid	7.63/7.42/7.62/6.49	(m)/(m)/(d, *J* = 16)/(d, *J* = 15.7)
	Caffeic acid	7.12/6.89/7.06	(d, *J*= 2)/(d, *J* = 8.2)/(dd, *J* = 8.4; 2)
	Sinapic acid	6.93/7.46/6.37/3.88	(s)/(d, *J* = 16)/(d, *J* = 16)/s
	p-coumaric acid	7.50/6.89/7.59?/6.49	(d, *J* = 8.49)/(d, *J* =8.2)
Others	Inositol	4.08/3.61/3.44/3.25	(t, *J* = 2.82)/(t, *J* = 9.97)/(dd, *J* = 10; 1.5?)/(t, *J* = 9.03)
	Nicotine	8.69/8.66/8.09/2.76	(d, *J* = 2.0)/(dd, *J* = 4.8; 1.4)/(dt, *J* = 4.7; 1.6)/(s)

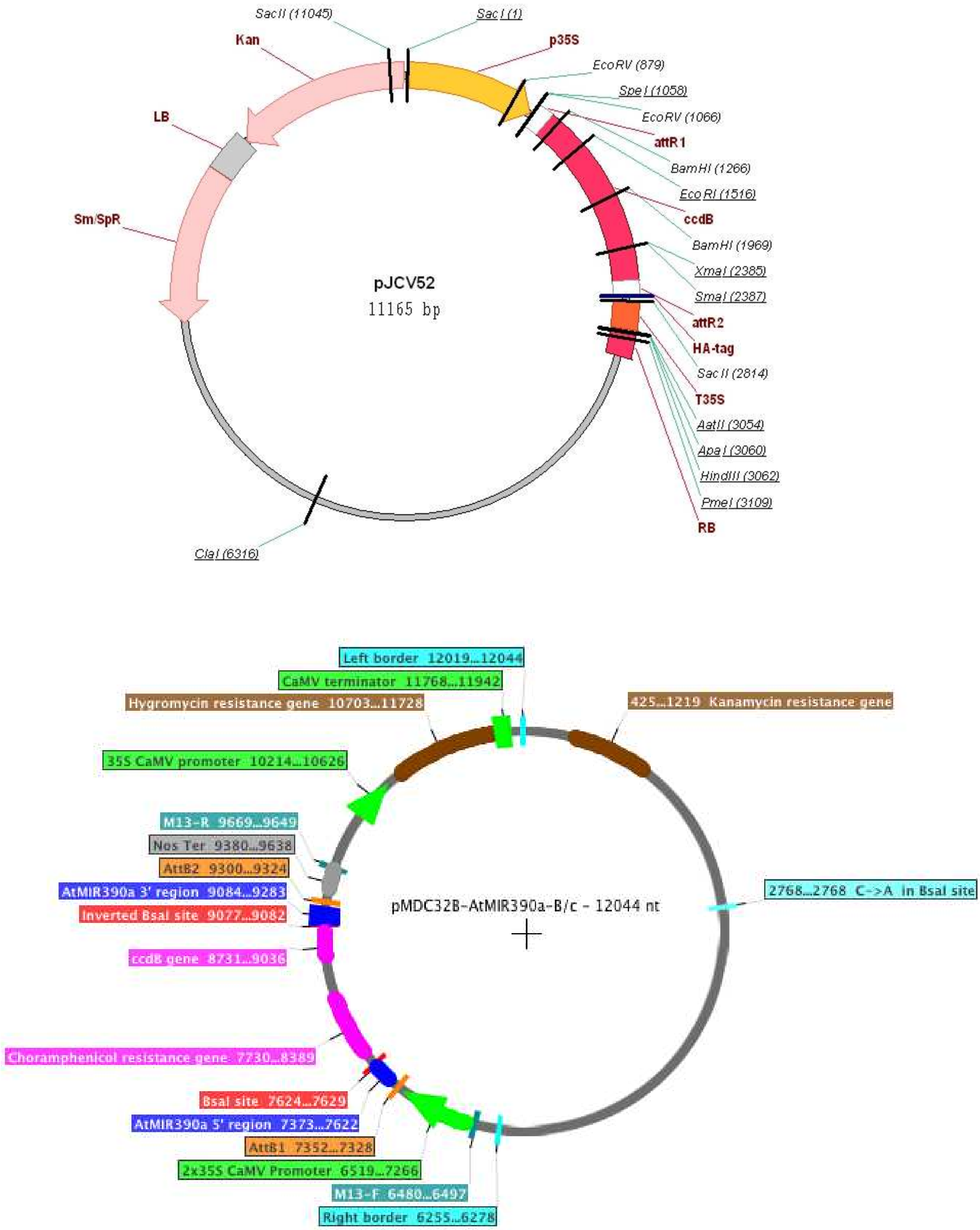

Fig. S1: Plant expression vectors pJCV52 by Karimi,et al.[5] and pMDC32B-AtMIR390a-B/c by Carbonell et al.[6]

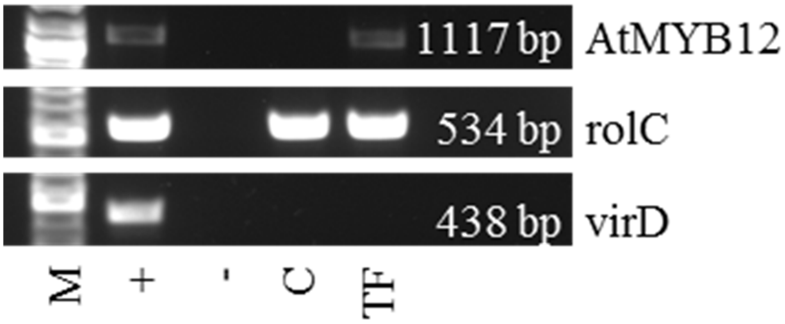

Fig. S2: PCR analysis of the genomic DNA of HR lines. (+) positive control (corresponding *A. rhizogenes* used for the infections), (-) negative control (DNA of *Nicotiana tabacum* wild type plant). C, wild type hairy root (control), TF, hairy root line carrying the AtMYB12 transcript

Supplementary information (1) for the design of amiRNA-CHS.

The amiRNA for *Nicotiana tabaccum* CHS, was designed from the gb|AF311783.1 sequence by the web tool: (http://p-sams.carringtonlab.org/amirna/designer) as described by Carbonell et al.[6]. The optimal amiRNA CHS sequence result was:

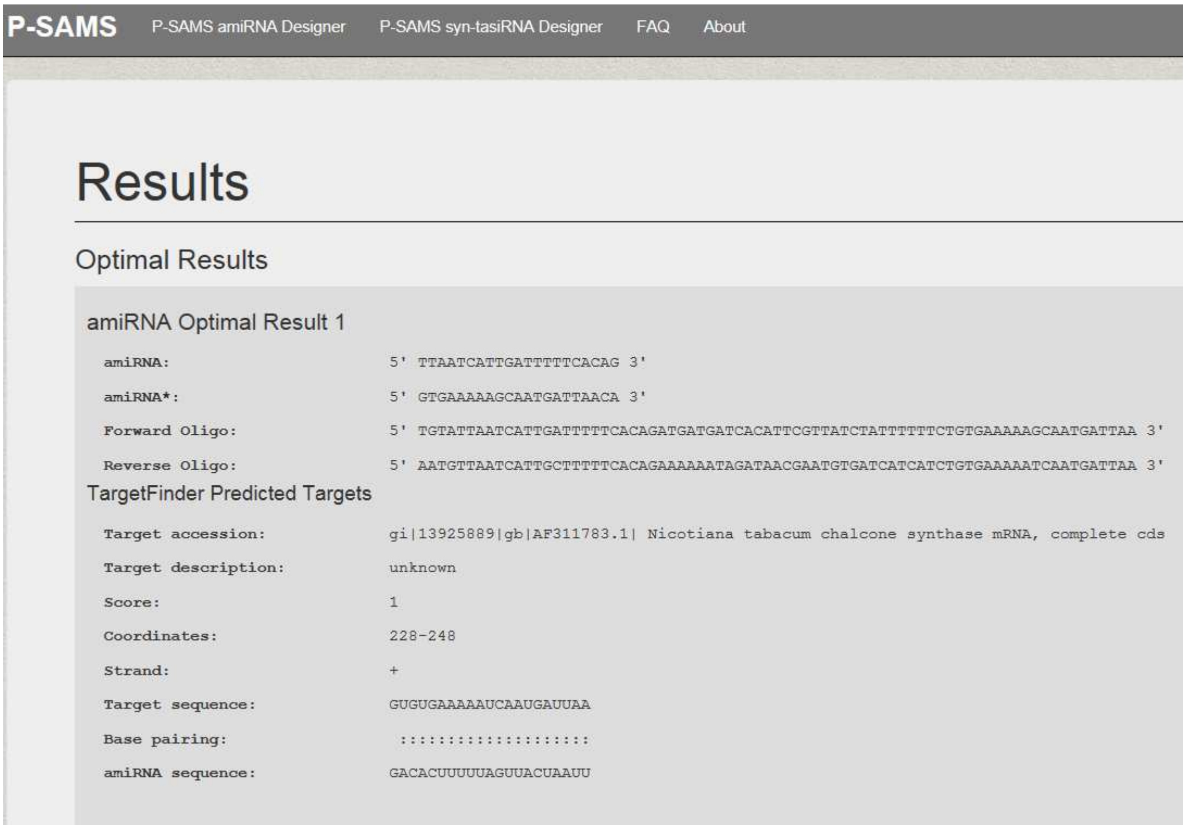

amiRNA optimal results:
amiRNA: 5' TTAATCATTGATTTTTCACAG 3'
amiRNA*: 5' GTGAAAAAGCAATGATTAACA 3'

Forward Oligo: 5'
TGTATTAATCATTGATTTTTCACAGATGATGATCACATTCGTTATCTATTTTTTC
TGTGAAAAAGCAATGATTAA 3'
Reverse Oligo: 5'
ATGTTAATCATTGCTTTTTCACAGAAAAAATAGATAACGAATGTGATCATCATC
TGTGAAAAATCAATGATTAA 3'

TargetFinder Predicted Targets
Target accession: gi|13925889|gb|AF311783.1| *Nicotiana tabacum* chalcone synthase mRNA, complete cds
Score: 1
Coordinates: 228-248

Strand: +

Target sequence:	GUGUGAAAAAUCAAUGAUUAA
Base pairing:	: : :: : : :: : : :: : : : :: : :: :
amiRNA sequence:	GACACUUUUUAGUUACUAAUU

Supplementary information (2). Sequence of *Nicotiana tabacum* chalcone synthase mRNA, complete cds

>gi|13925889|gb|AF311783.1| Nicotiana tabacum chalcone synthase mRNA, complete cds

ATCACTAGCCATTTGAAAACCCTAGTAATCGTCCATCATTTTTTCCGCAAAA
ATGGTGACCGTCGAGGAATTTCGTAGGGCGCAATGTGCCGAGGGTCCGGCC
ACGGTCATGGCTATCGGAACAGCCACACCTTCCAACTGTGTTGATCAAAGC
ACTTATCCTGATTATTATTTTCGTATCACTAATAGCGAGCATAAGGTTGAGC
TTAAGGAAAAATTTAAGCGCATGTGTGAAAAATCAATGATTAAGAAAAGGT
ACATGCACTTAACAGAGGAAATCTTGAAAGAGAATCCTAATATTTGTGCAT
ACATGGCACCTTCCCTTGATGCTAGACAAGACATAGTGGTGGTTGAAGTGC
CAAAACTTGGCAAAGAGGCAGCCCAAAAAGCCATCAAAGAATGGGGCCAG
CCCAAGTCCAAAATTACTCATTTGGTCTTTTGTACAACTAGTGGTGTAGACA
TGCCCGGGTGTGACTACCAACTCACTAAGCTACTCGGGCTCCGTCCATCGGT
CAAGCGGTTCATGATGTACCAACAAGGTTGCTTTGCCGGTGGCACGGTACTC
CGGATGGCTAAGGACTTGGCCGAAAACAACAAGGGCGCTCGAGTCCTTGTT
GTTTGTTCAGAGATCACCGCTGTCACGTTTCGTGGACCCAATGACACCCACT
TGGATAGTTTAGTTGGGCAAGCCCTTTTTGGTGATGGGGCAGCCGCGGTCAT
TATAGGTTCTGATCCAATTCCAGAGGTCGAGAGGCCTTTGTTCGAGCTTGTC
TCTGCAGCCCAAACTCTTCTCCCCGATAGCGAAGGCGCTATCGACGGTCACC
TTCGTGAAGTTGGGCTTACATTCCACTTACTCAAAGATGTTCCTGGGCTAAT
CTCAAAAAACATTGAGAAAAGCCTTGTGGAAGCA
TTCCAACCTTTGGGAATTTCTGATTGGAACTCTTTATTTTGGATTGCTCATCC
TGGTGGGCCTGCAATTTTGGACCAAGTTGAACTAAAATTGGGCCTAAAGCA
AGAGAAACTAAAGGCTACAAGAAAAGTATTAAGTAACTATGGCAACATGTC
TAGTGCTTGTGTGTTGTTTATTTTGGATGAAATGAGGAAAGCCTCTGCAAAA
GAAGGTTTGGGAACTACTGGTGAAGGGCTTGAATGGGGTGTGCTTTTTGGA
TTTGGGCCTGGGCTTACAGTTGAGACTGTTGTTCTCCACAGTGTTGCTACTT
AGTGGGCTTGGGCTTATATTGTGGGAAGATTTTAAGTGTTATAATTGTTTAT
TTTGTTTCTTGTGGTTGAATTTATTTTGTTTGTAATGAATGTATTTGCTCTATT
TTGCTATTTCATCTTGCAAATAATGAAATTTGTAATGTGAACTATTTAATCA
AAGAACTGAATTTCTTTCTCTT

Supplementary information (3). Sequence of *Vitis vinifera* stilbene synthase clone

ATGGCTTCAGTTGAGGAATTTAGAAACGCTCAACGTGCCAAGGGTCCGGCCA
CCATCCTAGCCATTGGCACAGCTACCCCCGACCACTGTGTCTACCAGTCTGAT
TATGCTGATTACTATTTCAGGGTCACTAAGAGCGAGCACATGACTGAGTTGAA
GAAGAAGTTCAATCGCATATGTGACAAATCAATGATCAAGAAGCGTTACATTC
ACTTGACCGAAGAAATGCTTGAGGAGCACCCAAACATTGGTGCTTATATGGC
TCCATCTCTTAACATACGCCAAGAGATTATTACTGCTGAGGTACCTAGACTTG
GTAGGGATGCAGCATTGAAGGCTCTTAAAGAGTGGGGCCAACCAAAGTCCA
AGATCACCCATCTTGTATTTTGTACAACCTCCGGTGTAGAAATGCCCGGTGCA
GATTACAAACTCGCTAATCTCTTAGGTCTTGAAACATCCGTTAGAAGGGTGAT
GTTGTACCATCAAGGGTGCTATGCAGGTGGAACTGTCCTTCGAACTGCTAAG
GATCTTGCAGAAAATAATGCAGGAGCACGAGTTCTTGTGGTATGCTCTGAGA
TCACTGTTGTTACATTCCGTGGGCCTTCCGAAGATGCTTTGGACTCTTTAGTT
GGCCAAGCCCTTTTTGGTGATGGGTCTTCAGCTGTGATTGTTGGATCAGATCC
AGATGTCTCGATTGAACGACCACTCTTCCAACTTGTTTCAGCGGCCCAAACA
TTTATTCCTAATTCAGCAGGAGCCATTGCCGGAAACTTACGTGAGGTGGGGCT
CACTTTTCATTTGTGGCCCAATGTGCCTACTTTGATTTCTGAGAACATAGAGA
AATGTTTGACCCAGGCTTTTGACCCACTTGGTATTAGCGATTGGAACTCGTTA
TTTTGGATTGCTCACCCAGGTGGCCCTGCAATTCTTGATGCAGTTGAAGCAA
AACTCAATTTAGAGAAAAAGAAACTTGAAGCAACAAGGCACGTGTTAAGTG
AGTATGGTAACATGTCTAGTGCATGTGTGTTGTTTATTTTGGATGAGATGAGA
AAGAAATCCCTAAAAGGGGAAAAAGCCACCACAGGTGAAGGATTAGATTGG
GGAGTACTATTTGGTTTTGGACCTGGCTTGACCATCGAAACTGTTGTGCTGCA
TAGCGTTCCTACGGTTACAAATTAA

Supplementary information (4). Blast result for aligned sequences of VvSTS and NtCHS

Score	Expect	Identities	Gaps	Strand
493 bits(546)	5e-143	820/1170(70%)	26/1170(2%)	Plus/Plus

```
Query  62   GTAGAGGACATTAGAAACGCTCAACGTGCCAAGGGTCCGGCCACCATCCTAGCCATTGGC  121
            || |||||  || | |  || ||| ||||| |||||||||||||  || | || || ||
Sbjct  62   GTCGAGGAATTTCGTAGGGCGCAATGTGCCGAGGGTCCGGCCACGGTCATGGCTATCGGA  121

Query  122  ACAGCTACTCCCGACCACTGTGTCTACCAGTCTGACTATGCTGATTACTATTTCAGGGTC  181
            ||||| || ||   | |||||||  | ||       ||| ||||||| |||||  |  ||
Sbjct  122  ACAGCCACACCTTCCAACTGTGTTGATCAAAGCACTTATCCTGATTATTATTTTCGTATC  181

Query  182  ACTAAGAGCGAGCACATGACTGAGTTGAAGAAGAAATTCAATCGCATATGTGACAAATCA  241
            ||||| |||||||| | |  |||| | ||| | ||||| || ||||| ||||| ||||||
Sbjct  182  ACTAATAGCGAGCATAAGGTTGAGCTTAAGGAAAAATTTAAGCGCATGTGTGAAAAATCA  241

Query  242  ATGATCAAGAAGCGTTACATTCACTTGACCGAAGAAATGCTTG-AGGAGCACCCAAACAT  300
            ||||| |||||  | ||||| ||||| || || ||||| |||| | ||| | || || ||
Sbjct  242  ATGATTAAGAAAAGGTACATGCACTTAACAGAGGAAAT-CTTGAAAGAGAATCCTAATAT  300

Query  301  TGGTGCTTATATGGCTCCATCTCTTAACATACGACAAGAGATTATCACTGCTGAGGTACC  360
            | |||| || ||||| || || ||| |     ||||||| ||  |    | ||| || ||
Sbjct  301  TTGTGCATACATGGCACCTTCCCTTGATGCTAGACAAGACATAGTGGTGGTTGAAGTGCC  360

Query  361  CAAACTTGGTAAAGAAGCAGCATTGAAGGCTCTTAAAGAGTGGGGCCAACCAAAGTCCAA  420
             |||||||| ||||| |||||    || ||  | ||||| |||||||| || ||||||||
Sbjct  361  AAAACTTGGCAAAGAGGCAGCCCAAAAAGCCATCAAAGAATGGGGCCAGCCCAAGTCCAA  420

Query  421  GATCACCCATCTTGTATTTTGTACAACCTCCGGTGTTGAAATGCCCGGTGCGGATTACAA  480
             || || ||| | || |||||||||||    ||||| || ||||||||    || ||| |
Sbjct  421  AATTACTCATTTGGTCTTTTGTACAACTAGTGGTGTAGACATGCCCGGGTGTGACTACCA  480

Query  481  ACTCGCTAATCTCTTAGGTCTCGAAACATCAGTTAGAAGAGTGATGTTGTACCATCAAGG  540
            |||| |||| ||  | || |||    |||| || |   |  | ||| ||||||| |||||
Sbjct  481  ACTCACTAAGCTACTCGGGCTCCGTCCATCGGTCAAGCGGTTCATGATGTACCAACAAGG  540

Query  541  GTGCTATGCAGGTGGAACTGTCCTCCGAACCGCTAAGGATCTTGCAGAGAATAATGCAGG  600
             |||| ||| ||||| || || ||||| |  ||||||||  | || || || ||    ||
Sbjct  541  TTGCTTTGCCGGTGGCACGGTACTCCGGATGGCTAAGGACTTGGCCGAAAACAACAAGGG  600
```

```
Query  601   AGCACGAGTTCTTGTGGTGTGCTCTGAGATCACTGTTGTTACATTTCGTGGGCCTTCCGA  660
              || ||||| ||||| || || || |||||||| | ||| || |||||||| |   || |
Sbjct  601   CGCTCGAGTCCTTGTTGTTTGTTCAGAGATCACCGCTGTCACGTTTCGTGGAC---CCAA  657

Query  661   AGATGC---TTTGGACTCTTTAGTTGGCCAAGCCCTTTTTGGTGATGGGTCTGCAGCTGT  717
              ||  |    |||||   ||||||||| ||||||||||||||||||||| | || || ||
Sbjct  658   TGACACCCACTTGGATAGTTTAGTTGGGCAAGCCCTTTTTGGTGATGGGGCAGCCGCGGT  717

Query  718   GATCGTTGGATCGGATCCAGATATCTCA-ATTGAACGACCACTTTTCCAGCTCGTCTCAG  776
              ||  | || || |||||| ||  |  |  | ||  | ||  | ||| |||| ||||| |
Sbjct  718   CATTATAGGTTCTGATCCA-ATTCCAGAGGTCGAGAGGCCTTTGTTCGAGCTTGTCTCTG  776

Query  777   CAGCCCAAACGTTTATTCCTAATTCAGCAGGTGCCATTGCCGGAAACTTGCGTGAGGTGG  836
             ||||||||||  || | ||  ||   | ||| || || | |||  || | ||||| || |
Sbjct  777   CAGCCCAAACTCTTCTCCCCGATAGCGAAGGCGCTATCGACGGTCACCTTCGTGAAGTTG  836

Query  837   GACTCACCTTTCATTTGTGGCCCA---ATGTGCCTACTTTAATTTCTGAGAACATAGAGA  893
             | || || || || ||    | ||   |||| |||    |||| ||  | ||||| ||||
Sbjct  837   GGCTTACATTCCACTT---ACTCAAAGATGTTCCTGGGCTAATCTCAAAAAACATTGAGA  893

Query  894   AATG-CTTGACTAAGGCTTTTGACCCACTTGGCATTAGCGATTGGAACTCGTTATTTTGG  952
             || | ||||   || || ||  | ||  | || |||   ||||||||||| |||||||||
Sbjct  894   AAAGCCTTGTGGAA-GCATTCCAACCTTTGGGAATTTCTGATTGGAACTCTTTATTTTGG  952

Query  953   ATTGCTCACCCAGGTGGCCCTGCAATTCTCGATGCAGTTGAAGCAAAACTCAATTTAGAG
1012

             |||||||| || ||||| ||||||||| | ||   |||||||  |||| |     || ||

Sbjct  953   ATTGCTCATCCTGGTGGGCCTGCAATTTTGGACCAAGTTGAACTAAAATTGGGCCTAAAG
1012

Query  1013  AAAAAGAAACTCGAAGCAACTAGGCATGTTTTAAGTGAGTACGGTAACATGTCAAGTGCA
1072

              || |||||||  | || || ||  | || |||||| | || || |||||||| |||||

Sbjct  1013  CAAGAGAAACTAAAGGCTACAAGAAAAGTATTAAGTAACTATGGCAACATGTCTAGTGCT
1072

Query  1073  TGTGTATTGTTTATTTTGGATGAGATGAGAAAGAAATCCT---CAAAAGGGGAAAAGGCT
1129

             ||||| ||||||||||||||||| |||||   |||| |||   ||||||  |    ||

Sbjct  1073  TGTGTGTTGTTTATTTTGGATGAAATGAG---GAAAGCCTCTGCAAAAGAAGGTTTGGGA
1129

Query  1130  ACCACAGGTGAAGGATTGGATTGGGGAGTATTATTTGGTTTTGGGCC-GGGCTTGACCAT
1188

             || || ||||||||  | || ||||| ||  | ||||| |||||||| |||||| ||  |
```

```
Sbjct  1130  ACTACTGGTGAAGGGCTTGAATGGGGTGTGCTTTTTGGATTTGGGCCTGGGCTT-ACAGT
1188

Query  1189  CGAAACTGTTGTGTTGCATAGCATTCCTAC  1218
              || ||||||||  | || ||  || ||||
Sbjct  1189  TGAGACTGTTGTTCTCCACAGTGTTGCTAC  1218
```

Unprocessed original scans for all of the blots in figure 2

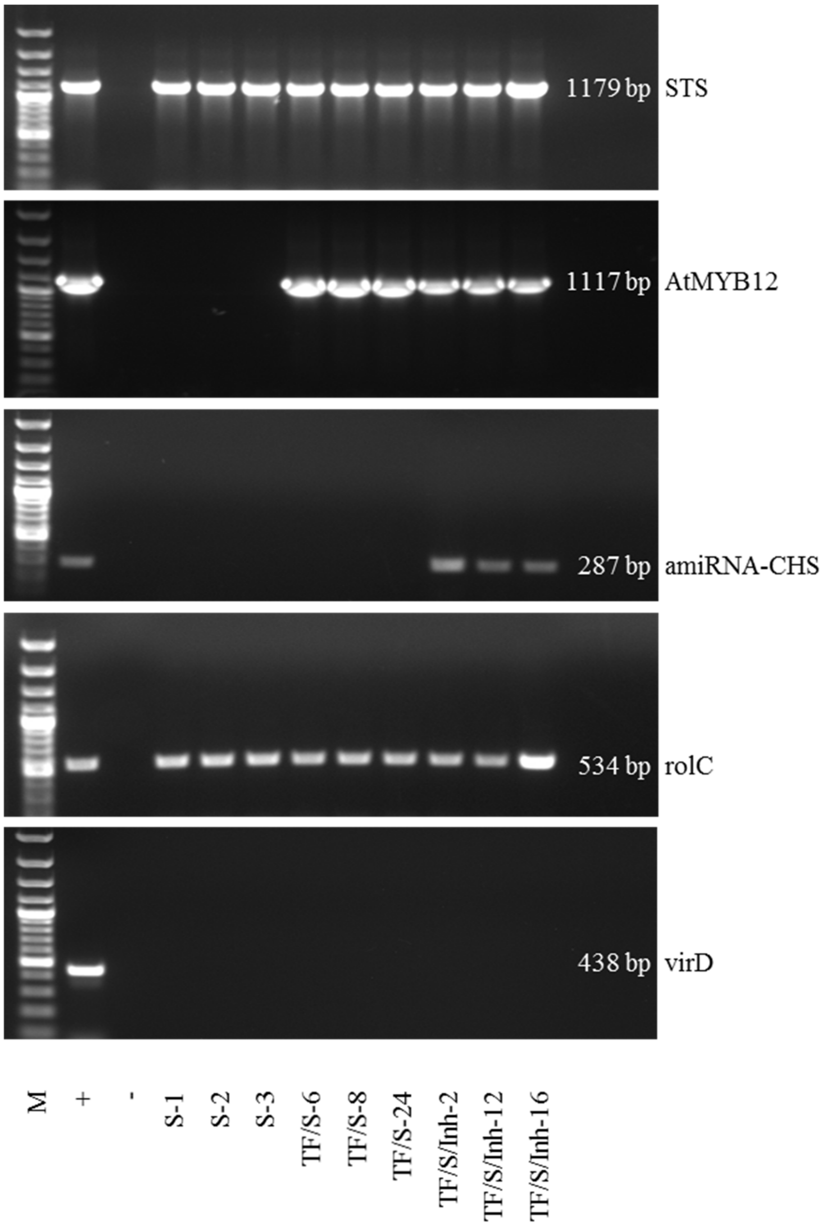

References

1. Hidalgo, D. *et al. Silybum marianum* cell cultures stably transformed with *Vitis vinifera* stilbene synthase accumulate t-resveratrol in the extracellular medium after elicitation with methyl jasmonate or methylated β-cyclodextrins. *Eng. Life Sci.* **17**, 686-694 (2017)

2. Slightom, J.L., Durand-Tardif, M., Jouanin, L. and Tepfer, D. Nucleotide sequence analysis of TL-DNA of *Agrobacterium rhizogenes* agropine type plasmid. *J, Biol. Chem.* **261**, 108-121 (1986).

3. Hirayama, T., Muranaka, T., Ohkawa, H. and Oka, A. Organization and characterization of the virCD genes from *Agrobacterium rhizogenes*. *Mol. Gen. Genet.* **213**, 229-237 (1988).

4. Hidalgo, D., *et al.* Bioconversion of stilbenes in genetically engineered root and cell cultures of tobacco. *Sci Rep.* **27**, 7:45331 (2017).

5. Karimi, M., Inzé, D. and Depicker, A. Gateway vectors for *Agrobacterium*-mediated plant transformation. *Trends Plant Sci.* **7**(5), 193-195 (2002).

6. Carbonell, A. *et al.* New Generation of Artificial MicroRNA and Synthetic Trans-Acting Small Interfering RNA Vectors for Efficient Gene Silencing in *Arabidopsis*. *Plant Physiol.* **165**(1):15-29 (2014).
